# Blended care vs. usual care in the treatment of depressive symptoms and disorders in general practice [BLENDING]: study protocol of a non-inferiority randomized trial

**DOI:** 10.1186/s12888-017-1376-1

**Published:** 2017-06-13

**Authors:** Btissame Massoudi, Marco H. Blanker, Evelien van Valen, Hans Wouters, Claudi L. H. Bockting, Huibert Burger

**Affiliations:** 1 Department of General Practice, University of Groningen, University Medical Center Groningen, PO Box 196, 9700 AD Groningen, the Netherlands; 2 Department of Clinical Psychology, University of Utrecht, Utrecht, The Netherlands; 3 Department of Clinical Psychology and Experimental Psychopathology, University of Groningen, Groningen, The Netherlands

**Keywords:** eHealth, Blended care, Internet-based treatment, Depression, Depressive symptoms, Depressive disorders, General practice, Primary care, Effectiveness, Cost-effectiveness

## Abstract

**Background:**

The majority of patients with depressive disorders are treated by general practitioners (GPs) and are prescribed antidepressant medication. Patients prefer psychological treatments but they are under-used, mainly due to time constraints and limited accessibility. A promising approach to deliver psychological treatment is blended care, i.e. guided online treatment. However, the cost-effectiveness of blended care formatted as an online psychological treatment supported by the patients’ own GP or general practice mental health worker (MHW) in routine primary care is unknown. We aim to demonstrate non-inferiority of blended care compared with usual care in patients with depressive symptoms or a depressive disorder in general practice. Additionally, we will explore the real-time course over the day of emotions and affect, and events within individuals during treatment.

**Methods:**

This is a pragmatic non-inferiority trial including 300 patients with depressive symptoms, recruited by collaborating GPs and MHWs. After inclusion, participants are randomized to either blended care or usual care in routine general practice. Blended care consists of the ‘Act and Feel’ treatment: an eight-week web-based program based on behavioral activation with integrated monitoring of depressive symptomatology and automatized feedback. GPs or their MHWs coach the participants through regular face-to-face or telephonic consultations with at least three sessions. Depressive symptomatology, health status, functional impairment, treatment satisfaction, daily activities and resource use are assessed during a follow-up period of 12 months. During treatment, real-time fluctuations in emotions and affect, and daily events will be rated using ecological momentary assessment. The primary outcome is the reduction of depressive symptoms from baseline to three months follow-up. We will conduct intention-to-treat analyses and supplementary per-protocol analyses.

**Discussion:**

This trial will show whether blended care might be an appropriate treatment strategy for patients with depressive symptoms and depressive disorder in general practice.

**Trial registration:**

Netherlands Trial Register: NTR4757; 25 August 2014. http://www.trialregister.nl/trialreg/admin/rctview.asp?TC=4757. (Archived by WebCite® at http://www.webcitation.org/6mnXNMGef)

**Electronic supplementary material:**

The online version of this article (doi:10.1186/s12888-017-1376-1) contains supplementary material, which is available to authorized users.

## Background

Depressive disorders are common. Up to 15% of people in high-income countries experience a major depressive episode at least once in their lifetime [1, 2] Depressive disorders cause high personal and economic burden of disease through their effects on many aspects of life such as participation and functioning [3–5]

The majority of patients (65% to 80%) with mild and moderate depression are treated by general practitioners (GPs) [6–11]. GPs in many countries including the Netherlands are recommended to use a (collaborative) stepped care management approach in the treatment of depression with antidepressants appearing only in the second step [12, 13]. Nevertheless, in routine general practice an estimated 70% of cases are primarily treated with antidepressants worldwide [9, 14–16]. This is worrisome as there is increasing evidence that antidepressants, while bringing about side effects, are ineffective in patients with mild to moderate depressive disorders which are particularly common in general practice [17–19]. This contrasts with psychological treatments that have convincingly shown effectiveness in these patients [20]. Moreover, most patients prefer psychological treatment [21].

Clear barriers for the implementation of psychological treatment in general practice exist. First, most GPs have too little time and experience to offer psychological treatment themselves [22, 23]. Second, referral for psychological treatment is hampered by limited accessibility due to practical barriers, such as high costs of treatment, limited availability of trained professionals and transportation issues [22, 24, 25]. Next, patients may experience emotional barriers such as feared stigmatization [26].

Referral by GPs to web-based programs for the treatment of depression circumvents these problems effectively by providing direct availability of psychological treatment at home. Several systematic reviews demonstrated the effectiveness of online psychological treatment in the treatment of depression [27–29]. Interventions without therapist guidance, however, showed small mean effect size and low adherence. In contrast, web-based interventions guided by a therapist (blended care) displayed relatively large mean effect size and better adherence [27–29], even comparable to face-to-face psychological treatment [30, 31]. Better adherence to treatment is important also for psychological interventions, as it is associated with greater response to treatment [32]. Therefore, guidance seems essential in the online treatment of depression. Importantly, it can be delegated to less trained or less experienced professionals (i.e. task-sharing) [33].

In previous trials on blended care in general practice, experienced professionals such as clinical psychologists or psychiatrist guided the patients [34–42]. In none of these studies were participants guided by their own GP or mental health worker (MHW). This may impair the generalizability of the results of these studies to Dutch general practice or similar settings. MWHs support primary care in delivering good quality mental health care by providing brief and simplified psychological treatment, blended care or self-help [43, 44]

In previous studies mainly online psychological interventions based on cognitive behavioral therapy (CBT) have been studied [38, 42, 45–47]. In a recent study behavioral activation has been shown as effective and more cost-effective compared to CBT [48] and it has been experienced as less complicated and more accessible to patients and therapists [49]. MHWs can learn this technique relatively quickly and its effectiveness is comparable to behavioral activation delivered by more specialist staff with more training [49]. Therefore in this trial we evaluate a blended behavioral activation treatment (guided ‘Act and Feel’ treatment).

Blended care has been expected to reduce costs, because part of the psychological treatment is provided online. However, a limited number of studies assessed the cost-effectiveness of blended care in general practice [50, 51].

To our knowledge, this is the first study investigating the effectiveness and cost-effectiveness of a blended behavioral activation treatment for depressive symptoms and disorders as performed in routine general practice.

## Methods

### Study design and aims

This study is a pragmatic, two-arm non-inferiority randomized controlled trial with an economic evaluation in general practice. The primary aim is to study the short-term (i.e. three months) effectiveness of a blended behavioral activation treatment compared to care as usual (CAU) for patients with depressive symptoms or disorders in general practice. Secondary aims include the short- and long-term (i.e. 12 months) cost-effectiveness and effects of blended care on daily activities, treatment satisfaction, medication use and other related resource use. In addition, we will explore the effects of the blended behavioral activation treatment and CAU on the course of real-time experiences of emotion, affect and events over the day.

### Participants

Adults (18 years or over) with a GP diagnosis of depressive symptoms or a depressive disorder for whom the GP considers a next step in the treatment of depression will be included [12]. Patients who fulfill the following criteria will be excluded: a) severe depression requiring prompt referral to specialized mental health service according to the GP (e.g. acute suicidality); b) current/past severe mental illness: schizophrenia, psychotic episode, bipolar disorder, depression with psychotic features; c) current anxiety disorder as primary diagnosis; d) current substance abuse (alcohol, drugs); e) current psychological treatment for depression; such as (online) cognitive behavioral therapy; f) no access to internet; g) insufficient computer skills; h) insufficient proficiency of Dutch language.

### Study setting and recruitment

This trial will be conducted in approximately 45 general practices in the Netherlands, located both in cities and in villages. This is a closed trial, i.e. GPs and MHWs recruit participants among their registered patients only. Informed consent will be obtained from each patient. Baseline assessments include the Structured Clinical Interview for DSM-IV disorders I (SCID-I). Modules A (mood episode), D (mood disorders) and F (anxiety disorders) allowing for diagnosing depressive disorders and anxiety disorders. The before-mentioned exclusion criteria are verified using the screening questions of the SCID-I; if screened positive than the total concerning section is administered. A trained interviewer telephonically conducts the SCID. Agreement between telephonic and face-to-face assessment is excellent (90% agreement) [52].

### Randomization

#### Sequence generation and allocation

**Fig. 1:**
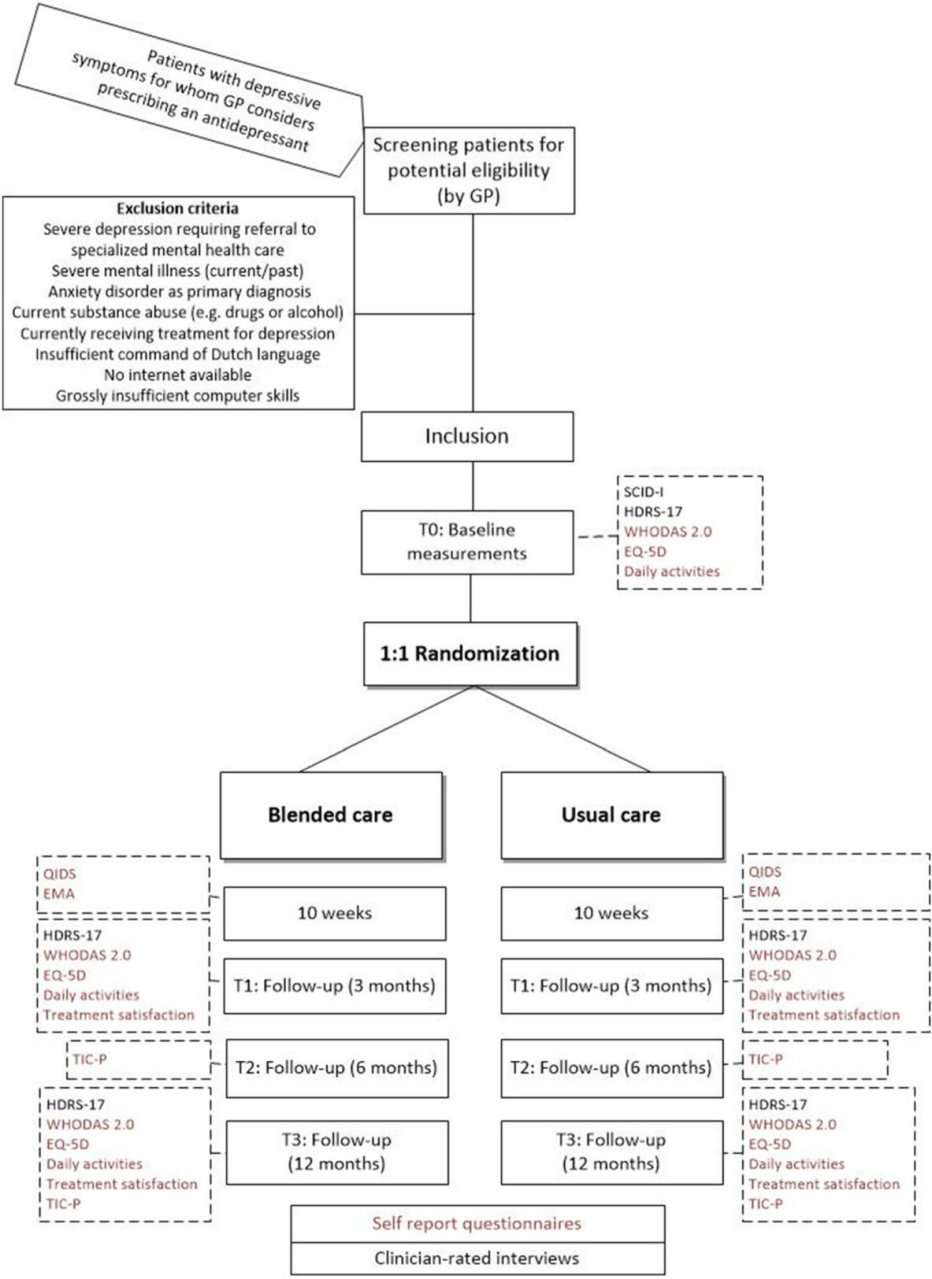
Outline of inclusion, baseline assessment, and randomization and follow-up assessmentsGP: general practitioner. SCID-I: Structured Clinical Interview for DSM disorders. HRSD-17: Hamilton Rating Scale for Depression, 17 items. EMA: ecological momentary assessments. QIDS-16: quick inventory of depressive symptomatology, 16 items. WHODAS-II: WHO Disability Schedule 2.0. EQ-5D: EuroQol five dimensions questionnaire. TIC-P: Trimbos and iMTA questionnaire on Costs associated with Psychiatric illness

After baseline assessment participants are 1:1 randomly assigned to one of the treatment groups, stratified by sex, diagnosis (depressive symptoms or depressive disorder) and general practice (availability of a MHW). The randomization sequence is computer-generated with randomly varying block sizes and is concealed by an independent research department. After randomization, the researchers inform the general practice about the treatment allocation. The GP/MHW shares this information with the participant during the follow-up consultation. This trial is single-blinded, i.e. the treatment allocation is concealed from the outcome assessor and participants are asked not to reveal the treatment allocation (Fig. 1).

### Guided ‘act and feel’ treatment

The guided ‘Act and Feel’ treatment is an online self-management program blended with direct contacts (face-to-face or telephonic) with a MHW [53, 54]. The content of Act and Feel is based on the face-to-face behavioral activation by Lewinsohn [55] and focuses on depressive symptoms. Behavioral activation is based on the behavioral theory stating that depressive behavior is a consequence of low rates of response-contingent positive reinforcement [55]. Therefore, behavioral activation concentrates on activating individuals by increasing activities that are potentially reinforcing for the individual. ‘Act and Feel’ consists of eight modules with integrated monitoring of depressive symptomatology (including suicidality) and automatized feedback in a secure online environment. First, it provides psycho-education about depression and the rationale behind behavioral activation. Second, participants are given insight in their daily mood and behavior/activities using daily activity mood monitoring. Preplanned potential pleasurable activities are mood-independent increased to increase the sense of satisfaction and enhance mood in the long term. There is additionally attention for the role of avoidant behavior in depression and how to turn this to proactive behavior. Finally, it concludes with the development of a personal relapse prevention strategy.

Every module has a fixed structure, starting with a visual analogue scale (VAS) mood assessment (on a Likert scale from 1 to 10); the rationale of that module and specific exercises and it concludes with a summary and evaluation of that module. The ‘Act and Feel’ treatment provides automatically produced graphs of self-ratings mood score and depressive symptomatology.

#### Guidance

The MHW supports the participants with consultations of a targeted 30 min at two, four and six weeks. The purposes of these consultations are to motivate and support the participants during the activation process and to enhance adherence. In addition, participants receive e-mail reminders when inactive on the online environment for two weeks. The MHWs who guide Act and Feel follow a one-day training session on guidance of Act and Feel and behavioral activation and on-demand supervision of cases by the psychologist research assistant. Before implementation of this treatment, the usability of the ‘Act and Feel’ treatment was evaluated in a pilot study with twenty healthy volunteers. By means of this feedback the treatment was adjusted and after adjustment retested in a second pilot.

### Care as usual

The control group receives CAU for depression, left at the discretion of the GP. Patients with depressive disorders in routine usual general practice care are offered mostly pharmacological treatment and receive alongside some psychological treatment, such as non-directive counseling [56, 57]. We will not interfere with CAU but document it carefully. Nevertheless, GPs (or delegated to the MHW) are asked to plan consultations (telephonic or face-to-face) with the participant at two, four and six weeks as recommended by the guideline of the Dutch College of GPs thus mirroring the intervention arm [12]. In addition, patients are monitored for depressive symptomatology and acute suicidality.

### Concomitant treatments

Given the trial’s pragmatic nature, the aim is to quantify the real life difference in the course of depressive symptoms between blended behavioral activation and usual care. Consequently, all participants in both arms are allowed to continue or to start any medical or non-medical concomitant treatment, except for psychological treatment, which we ask not to offer in the intervention group during the first three months. For both groups, the use of all forms of formal health care during the trial is carefully documented.

### Participant timeline and assessments

Both clinical and economical outcomes are measured (Table 1). Interviewer-rated assessments are performed telephonically (i.e. SCID-I and HRSD-17) and the online self-report assessments through an online environment (ResearchOnline®). Follow-up assessments are at 3, 6 and 12 months.

**Table 1:** Overview of the follow-up assessments of the participants

Measures	Description	Baseline	Week 1–10	3 months	6 months	12 months
HRSD-17	Depressive symptom rating	X		X		X
EMA	Daily real time ratings of emotion, affect and events		X			
SCID-I	Diagnosis of current depressive disorder	X				
QIDS-16	Depressive symptoms rating		X			
WHODAS-II	Functional impairment and activity limitation	X		X		X
EQ-5D	Health status	X		X		X
Daily activity section of Leidsche Rijn Health Questionnaire	Regular daily activities	X		X		X
Treatment satisfaction				X		X
TIC-P	Direct and indirect costs			X	X	X
Demographics	Socio-demographic characteristics	X				
Resource use	Medication use, number of referrals and GP consultations					X

HRSD-17: Hamilton Rating Scale for Depression, 17 items

EMA: ecological momentary assessments

SCID-I: Structured Clinical Interview for DSM disorders

QIDS-16: quick inventory of depressive symptomatology, 16 items

WHODAS-II: WHO Disability Schedule 2.0

EQ-5D: EuroQol five dimensions questionnaire

TIC-P: Trimbos and iMTA questionnaire on Costs associated with Psychiatric illness

GP: general practitioner

Random real-time assessments will be done in the first ten weeks of participation. In addition to the assessments, all patients are monitored for severe depression and acute suicidality by filling in the self-report questionnaire QIDS weekly during ten weeks.

### Outcomes

#### Primary outcome

The primary outcome is the reduction of depressive symptoms from baseline to three months follow-up, as measured with the Hamilton Depression Scale 17-items (HRSD-17) [58–60]. This is a clinician-rated scale consisting of 17 items. This scale has been widely used in clinical trials and serves as gold standard for assessment of depression. It has been found valid and reliable measurement outcome with a higher inter-rater reliability and test-retest reliability [59, 61].

### Secondary outcomes

#### Depression related outcomes

The reduction of depressive symptoms on the HRSD-17 is assessed from baseline to 12 months of follow-up. Additionally, response defined as a 50% reduction on the HRSD-17 is evaluated at 3 and 12 months. Furthermore, depressive symptoms are assessed weekly using the self-reported measurement Quick Inventory of Depressive symptoms self-reported 16 items (QIDS-SR 16). The QIDS-SR 16 are used to monitor depressive symptoms and to signal acute suicidality and severe depression during the first ten weeks of treatment. When exceeding the threshold of severe depression (i.e. HRSD-17 score 16 or higher) or acute suicidality (i.e. score of 2 or 3 on the suicidality item of the QIDS-R), all patients receives an automatic message to contact their GP and MHW and the researcher informs the GP.

#### Treatment satisfaction

Treatment satisfaction is assessed using a self-developed questionnaire, consisting of five items. Each question is scored on a Likert-type scale from 1 to 4. The questionnaire addresses overall service satisfaction; i.e. satisfaction with treatment, recommendation of treatment, satisfaction about effectiveness of treatment and willingness to repeat treatment if needed. This questionnaire is shown in Additional file 1.

#### Health status

General health status is assessed with the EuroQol (EQ-5D), which is a validated self-administered questionnaire that contains a descriptive section and a valuation section. The descriptive section consists of five dimensions: mobility, self-care, usual activities, pain/discomfort and anxiety/depression. Each of these five dimensions has three levels of severity: no problems, some/moderate problems and extreme problems. Values of the five dimensions are linearly transformed into one single score, ranging from 0 to 1 (lower scores represent more problems). The valuation section of the EQ-5D contains a visual analogue scale; this vertical visual scale ranges between ‘best imaginable health state = perfect health’ (score of 100) and ‘worst imaginable health state = death’ (score of 0). The EQ-5D valuation section has been shown to be efficient and easy to use [62].

#### Functional impairment and activity limitations

Functional impairment and activity limitations are assessed using the 12-item self-report World Health Organization Disability Assessment Schedule 2.0 (WHODAS 2.0). The WHODAS 2.0 is based on the conceptual framework of the International Classification of Functioning, Disability and Health (ICF). It provides a global measure of disability and 7 domain-specific scores. It assesses health and disability on six dimensions, namely cognition, mobility, self-care, getting along, life activities and participation. WHODAS 2.0 has been validated in 1119 patients across 7 European centers with 13 chronic conditions and it has been shown to have good metric properties [63]

#### Daily activities

Daily activities are assessed using part of the Health Questionnaire of the Utrecht Health project [64] This part of the questionnaire assesses daily activity level during a regular week in the past months, i.e. the kind and duration of these activities. The blended care treatment focuses on activating participants, by increasing their potential pleasurable activities. By assessing daily activities we study a potential prognostic mediating factor in the recovery of depression.

#### Ecological momentary assessment (EMA)

We will capture real-time momentary ratings of cognitions as well as affect and emotions with so-called Ecological Momentary Assessment (EMA). Compared to conventional questionnaires, potential advantages of prospective precise measurement with EMA are less recall bias and increased detection of vulnerable patients with adverse disease trajectories [65].

**Fig. 2:**
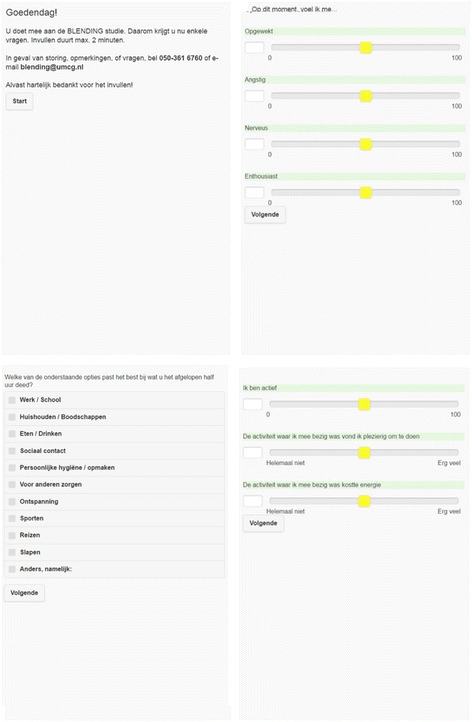
Screenshot from the ‘DE-free’ application

Specifically, using a smart phone application called ‘DE Free’ (Bockting, used software TEMPEST, Batalas & Markopoulos) (Fig. 2), auditory cue signals will be sent to participants’ cellphones. Thereupon, participants will be asked to rate their momentary states. These include an appraisal of positive affect (i.e. being cheerful, content, and energetic) and negative affect (i.e. feeling gloomy, nervous, insecure, lonely, irritated, helpless, and guilty), and activity levels (i.e. being active, pleasantness of activity, and energy involved in performing activity). Momentary states are rated on visual analogue scales, which are either numerical and ranging from 0 to 100 or ordinal and ranging from e.g. “not at all” to “very much”. Furthermore, participants will be asked to indicate the specific activities they were undertaking and the specific situation they were in while rating these momentary states (e.g. being at home and doing household chores). Participants will be asked to rate their momentary states in the following time schedule: every other day, 5 times per day, over the course of a 10-week period. Auditory signals will be sent at random moments during the following time intervals: equally spread between 8.30 a.m. and 7.30 p.m.

### Resource use

#### Costs related to the intervention

To calculate costs related to the internet treatment, we include costs of running the website platform and costs of the guidance by the GP or MHW.

#### Direct and indirect costs

We will measure health care costs in general (direct costs) as well as productivity losses (indirect costs) with the revised version of the Trimbos and iMTA Questionnaires on Costs Associated with Psychiatric Illness (TiC-P) [66, 67]. Direct costs are measured by investigating, which contact with health professionals has occurred and which type of medication has been prescribed. Indirect costs are measured by work absenteeism and reduced productivity. The TiC-P questions cover the previous six months; it is administrated at 6 and 12 months follow-up.

### Sample size

The sample size is based on demonstrating non-inferiority of blended care compared to usual care for the primary outcome, i.e. the reduction on HRSD-17 from baseline to three months follow-up. An acceptable non-inferiority margin has been defined as the maximum clinically irrelevant difference between treatments [68]. The non-inferiority margin is set to be the half of minimal clinical important difference of 3 points on the HRSD-17 scale as advised by the FDA [60, 69, 70]; i.e. a non-inferiority margin of 1.5 points at the HRSD-17 (0.31 SD). The sample size was calculated with one-sided t-test with a power of 80% (α = 0.025) to detect a non-inferiority margin of 1.5. We compensated for an expected loss to follow-up of 20%. We adjusted for covariance, as advised to reduce required sample sizes and costs [71, 72]. In this correction we assumed a correlation coefficient (ρ) of 0.5, as recommended when the correlation coefficient is unknown [71, 73]. The number of participants needed is then corrected with design factor (1 − *ρ* ^2^), where ρ is the correlation coefficient. This resulted in a calculated design factor of 0.75. The final number calculated necessary to complete the trial is 151 participants per condition (a total of *N* = 302).

### Statistical methods

First, we will compare demographic characteristics of the trial population, to assure whether the randomization procedure has been successful.

The principle analysis will follow an intention-to-treat (ITT) strategy including all patients analyzed according to their randomized group. Supplementary analyses will be done on a ‘per protocol’ basis, meaning that they will be restricted to those participants that completed a minimum of three sessions in the intervention group.

The primary outcome, i.e. reduction in symptoms of depression from baseline to the 3 months follow-up, will be analyzed using a linear regression approach to analysis of covariance (ANCOVA) with the follow-up value of the outcome as the dependent variable and treatment and baseline value of the outcome as the independent variables. Secondary outcomes that were measured more than twice will be analyzed using linear mixed models for fixed (e.g. treatment) and random effects (e.g. patient). These models are superior for the analysis of longitudinally correlated data and can optimally deal with missing values [71]. In these analyses, a treatment*time variable indicating the magnitude of the effect of the intervention on the change of the outcome variable will be included as an independent variable. If important baseline differences exist in prognostic important variables, despite randomization, we will adjust the analyses by including variables as covariates in additional analyses. Potential baseline differences will be reexamined before the per protocol analyses. Dichotomous outcome variables measured more than twice will be analyzed using generalized linear mixed models (mixed-effects logistic regression).

All effect parameters will be supplied with a 95% confidence interval. Statistical significance level will be set at 0.025, one-sided, as appropriate with the chosen non-inferiority design.

EMA data of real time momentary ratings will be examined in a bivariate vector-autoregressive Bayesian dynamic analysis and related analytic approaches to model the time structure of the data [Krone in preparation 2016; Krone submitted]. Feasibility and tolerability of EMA assessment by participants will be examined by assessing how many participants will be able to complete at least 70% of the EMA ratings. Based on the literature we will exclude participants with 30% or more missing on the EMA ratings (Krone in preparation, [74, 75]).

### Additional analysis

#### Economic outcomes

The cost-effectiveness and cost-utility of blended care will be studied from a societal perspective, using a time horizon of 12 months. To determine the cost-effectiveness of both treatments, we will estimate costs in both treatment groups. To calculate the total direct medical costs we will estimate the resource use. The resource use will be valued according to the Dutch guidelines for cost studies [67]. Costs of blended care will be estimated by true resources used. Medication use will be valued based on costs a provided by The National Health Care Institute (Zorginstituut Nederland, ZIN). Total costs for both interventions will be summed-up over the 12-month study period. An incremental cost effectiveness ratio (ICER) will be calculated using the primary measure of effect: HDRS-17. In addition, a secondary analysis will be performed using the number of patients with a response as defined under secondary outcomes. In order to be able to compare the effects of blended care to other health care interventions, also a cost-utility analysis will be done; using EQ-5D defined utilities. Bootstrapping will be performed to depict the 95% confidence intervals surrounding the cost effectiveness, and cost utility ratios. Cost-effectiveness planes will be plotted in order to represent the cost-effectiveness graphically.

## Discussion

There is a need to investigate accessible ways to offer psychological treatment in general practice as both GPs and patients prefer psychological treatment [21–23]. However, barriers to psychological treatment in general practice such as time constraints and lack of experience of the GP (28–30), result in GPs prescribing pharmacological treatment even as a first step [76]. This contrasts sharply with the guidelines recommending non-pharmacological treatment as the first step [12, 13] and is unwanted in view of the side effects, poor adherence, and long-term use after remission associated with antidepressant use [77–79]. Blended care interventions may provide GPs a feasible instrument to offer structured psychological treatment.

The current paper presents the design of a pragmatic non-inferiority trial to study the effectiveness and cost-effectiveness of a blended behavioral activation treatment for depressive symptoms and disorders in general practice.

While previous studies mainly used online psychological interventions based on cognitive behavioral therapy (CBT) [28, 32, 80], behavioral activation has been shown as effective and more cost-effective compared to CBT(50). In addition, it has been experienced as less complicated and more accessible to patients and therapists [81]. MHWs can learn the technique required quickly and its effectiveness is comparable to behavioral activation delivered by more specialist staff with more training [49]. It is expected that blended behavioral activation will provide an accessible and easier way to provide psychological treatment in general practice.

To our knowledge, this is the first trial of blended care based on behavioral activation in general practice provided by patients’ own GP or MHWs including assessments of real-time experiences during treatment by the EMA. An important strength of this trial is that it is conducted in routine general practice. Patients are recruited and coached by their own GPs and MHWs during treatment, hereby mirroring routine general practice and increasing the study’s generalizability. Another strength is that the EMA has the advantage of reducing memory distortions compared to conventional questionnaires. It gives insight into actual experiences during treatment [82]. In future, knowledge of patients’ real-time experiences may enable the development of more personalized blended care for depression [65]. This randomized controlled trial will show whether a blended behavioral activation treatment is a non-inferior treatment strategy for patients with depressive symptoms and depressive disorder in general practice.

## Additional file

Additional file 1: Questionnaire overall service satisfaction; i.e. satisfaction with treatment, recommendation of treatment, satisfaction about effectiveness of treatment and willingness to repeat treatment if needed. (DOCX 1224 kb)

## Abbreviations

CAU: Care as usual
CBT: Cognitive behavioral therapy
EMA: Ecological Momentary Assessments
EQ-5D: EuroQol five dimensions questionnaire
FDA: Federal Drug Administration
GP: General practitioner
HRSD-17: Hamilton Rating Scale for Depression, 17 items
ICER: Incremental Cost-Effectiveness Ratio
ICF: International Classification of Functioning, Disability and health
ITT: Intention To Treat
MHW: Mental health worker
QIDS-16: Quick inventory of depressive symptomatology, 16 items
SCID: Structured Clinical Interview for DSM disorders
TIC-P: Trimbos and iMTA questionnaire on Costs associated with Psychiatric illness
VAS: Visual analogue scale
WHODAS-II: WHO Disability Schedule 2.0; EQ-5D
