# Supplementary figures and images for: Blended care vs. usual care in the treatment of depressive symptoms and disorders in general practice [BLENDING]: study protocol of a non-inferiority randomized trial
Source: BMC Psychiatry. 2017 Jun 13;17:218. doi: 10.1186/s12888-017-1376-1 (PMC5470276; doi:10.1186/s12888-017-1376-1)

Additional file 1

Questionnaire overall service satisfaction


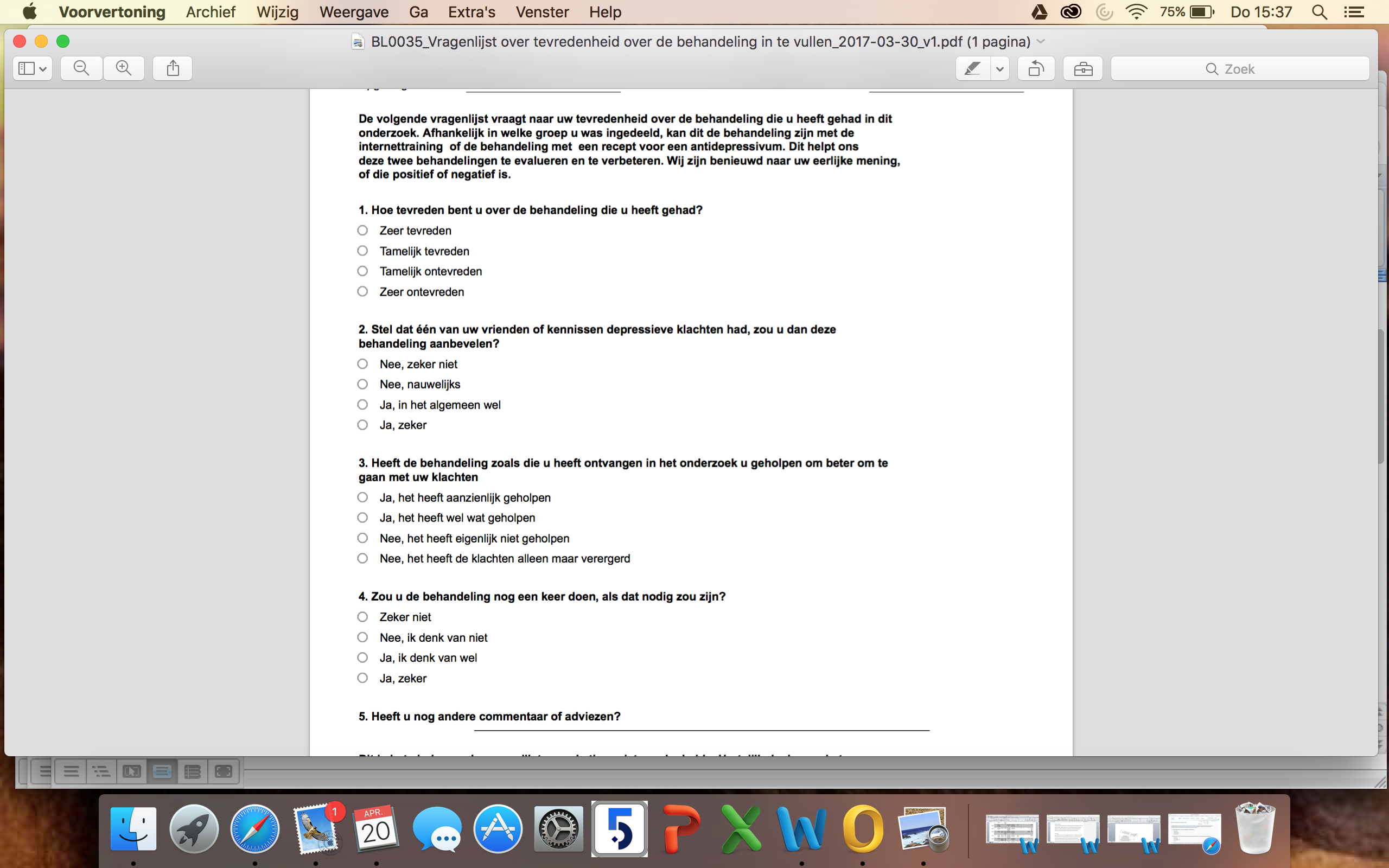

Supplement: Additional file 1: — Questionnaire overall service satisfaction; i.e. satisfaction with treatment, recommendation of treatment, satisfaction about effectiveness of treatment and willingness to repeat treatment if needed. (DOCX 1224 kb) [file 12888_2017_1376_MOESM1_ESM.docx]
